# Center effect in intubation risk in critically ill immunocompromised patients with acute hypoxemic respiratory failure

**DOI:** 10.1186/s13054-019-2590-7

**Published:** 2019-09-06

**Authors:** Guillaume Dumas, Alexandre Demoule, Djamel Mokart, Virginie Lemiale, Saad Nseir, Laurent Argaud, Frédéric Pène, Loay Kontar, Fabrice Bruneel, Kada Klouche, François Barbier, Jean Reignier, Annabelle Stoclin, Guillaume Louis, Jean-Michel Constantin, Florent Wallet, Achille Kouatchet, Vincent Peigne, Pierre Perez, Christophe Girault, Samir Jaber, Yves Cohen, Martine Nyunga, Nicolas Terzi, Lila Bouadma, Christine Lebert, Alexandre Lautrette, Naike Bigé, Jean-Herlé Raphalen, Laurent Papazian, Dominique Benoit, Michael Darmon, Sylvie Chevret, Elie Azoulay

**Affiliations:** 1 Medical Intensive Care Unit, Saint-Louis Teaching Hospital, APHP, Paris, France; 2 ECSTRA team, Biostatistics and Clinical Epidemiology, UMR 1153 (Center of Epidemiology and Biostatistic Sorbonne Paris Cité, CRESS), INSERM, Paris Diderot University, Paris, France; 3 Groupe Hospitalier Pitié-Salpêtrière Charles Foix, Service de Pneumologie, Médecine Intensive et Réanimation (Département R3S), AP-HP, INSERM, UMRS1158 neurophysiologie respiratoire expérimentale et clinique, Sorbonne Université, Paris, France; 4 Intensive Care Unit, IPC, Marseille, France; 5 Critical Care Center, CHU de Lille, Lille, France; 6 Medical Intensive Care Unit, Edouard Herriot Teaching Hospital, Lyon, France; 7 Medical Intensive Care Unit, Cochin Teaching Hospital, Paris, France; 8 Critical Care Center, Centre Hospitalier Universitaire-Amiens, Amiens, France; 9 Intensive Care Unit, Hôpital Andre Mignot-Le Chesnay, Paris, France; 10 Intensive Care Unit, Lapeyronie University Hospital, Montpellier, France; 11 Medical Intensive Care Unit, La Source Hospital-CHR Orleans, Orléans, France; 12 Réanimation Médicale, Centre Hospitalier Universitaire-Nantes, Nantes, France; 13 Critical Care Center, Institut Gustave Roussy, Villejuif, France; 14 Intensive Care Unit, CHR de Metz-Thionville, Metz, France; 15 Department of Anesthesiology and Critical Care, Groupe Hospitalier Pitié-Salpêtrière Charles Foix, Paris, France; 16 Medical Intensive Care Unit, Hôpital Lyon-Sud, Lyon, France; 17 Medical Intensive Care Unit, Angers Teaching hospital, Angers, France; 18 Intensive Care Unit, Centre Hospitalier Métropole-Savoie, Chambery, France; 19 Medical Intensive Care Unit, Brabois University Hospital, Nancy, France; 20 Medical Intensive Care Unit, Hôpital Charles Nicolle, Rouen, France; 21 Critical Care Center, CHRU Montpellier-Saint-Eloi, Montpellier, France; 22 Intensive Care Unit, Hôpital d’Avicenne, APHP, Bobigny, France; 23 Medical Intensive Care Unit, Victor Provo Hospital, Roubaix, France; 24 Medical Intensive Care Unit, CHU de Grenoble Alpes, Grenoble, France; 25 Medical Intensive Care Unit, CHU Bichat, Paris, France; 26 Intensive Care Unit, Centre Hospitalier Départemental Les Oudairies, La Roche-Sur-Yon, France; 27 Medical Intensive Care Unit, Gabriel-Montpied University Hospital, Clermont-Ferrand, France; 28 Medical Intensive Care Unit, Hôpital Saint-Antoine, Paris, France; 29 Department of Anesthesia and Critical Care, Hôpital Necker, Paris, France; 30 Réanimation DRIS, Hôpital Nord, Marseille, France; 31 Medical ICU, Ghent University Hospital, Ghent, Belgium

**Keywords:** Center effect, Intubation, Neutropenia, Leukemia, Hypoxemia

## Abstract

**Background:**

Acute respiratory failure is the leading reason for intensive care unit (ICU) admission in immunocompromised patients, and the need for invasive mechanical ventilation has become a major clinical endpoint in randomized controlled trials (RCTs). However, data are lacking on whether intubation is an objective criteria that is used unbiasedly across centers. This study explores how this outcome varies across ICUs.

**Methods:**

Hierarchical models and permutation procedures for testing multiple random effects were applied on both data from an observational cohort (the TRIAL-OH study: 703 patients, 17 ICUs) and a randomized controlled trial (the HIGH trial: 776 patients, 31 ICUs) to characterize ICU variation in intubation risk across centers.

**Results:**

The crude intubation rate varied across ICUs from 29 to 80% in the observational cohort and from 0 to 86% in the RCT. This center effect on the mean ICU intubation rate was statistically significant, even after adjustment on individual patient characteristics (observational cohort: *p* value = 0.013, median OR 1.48 [1.30–1.72]; RCT: *p* value 0.004, median OR 1.51 [1.36–1.68]). Two ICU-level characteristics were associated with intubation risk (the annual rate of intubation procedure per center and the time from respiratory symptoms to ICU admission) and could partly explain this center effect. In the RCT that controlled for the use of high-flow oxygen therapy, we did not find significant variation in the effect of oxygenation strategy on intubation risk across centers, despite a significant variation in the need for invasive mechanical ventilation.

**Conclusion:**

Intubation rates varied considerably among ICUs, even after adjustment on individual characteristics.

**Electronic supplementary material:**

The online version of this article (10.1186/s13054-019-2590-7) contains supplementary material, which is available to authorized users.

## Background

There is an increasing trend to consider mortality as a sub-optimal primary endpoint in randomized controlled trials (RCTs) involving critically ill patients [1–4]. In acute respiratory failure, the need for invasive mechanical ventilation may appear as a legitimate alternative to avoid negative RCTs. Indeed, over the last two decades, strategies to avoid invasive mechanical ventilation and related complications have been evaluated in patients with acute hypoxemic respiratory failure [1, 3, 5–7]. This consideration is even more relevant in immunocompromised patients in whom higher mortality rates have been reported in patients requiring invasive mechanical ventilation, most particularly in those meeting criteria for acute respiratory distress syndrome [8, 9]. Thus, noninvasive ventilation (NIV), continuous positive airway pressure (cPAP), and the use of high-flow nasal cannula (HFNC) have been widely explored in this subset of patients [1, 3, 5–7]. Hence, the need for intubation has become a major clinical endpoint in RCTs of patients with acute respiratory failure [8, 10–14].

In both cohort studies and RCTs, invasive mechanical ventilation delivery may however vary across centers, possibly leading to biased conclusions. This heterogeneity in mechanical ventilation use refers to “center effects,” a concern already explored in the intensive care setting [15]. Actually, center effects can be divided into two components: (i) heterogeneity in the distribution of the study endpoint across centers and (ii) heterogeneity in the treatment effect on the outcome across centers known as “treatment by center interaction.” To date, no study has investigated a potential center effect in studies of immunocompromised patients with hypoxemic acute respiratory failure. In the present study, we sought to identify a center effect about the need for endotracheal intubation in immunocompromised patients with acute respiratory failure.

## Methods

### Centers and patients

This study included adult immunocompromised patients with hypoxemic acute respiratory failure (ARF) from the TRIAL-OH cohort and the HIGH randomized controlled trial (RCT). Details of each study have been described previously [3, 16]. Firstly, we derived from the TRIAL-OH study (1011 patients, 17 centers, 2010–2011) [16] a cohort of hematological patients admitted to ICU for ARF. We selected patients with the following criteria: ARF as defined by tachypnea > 30/min, respiratory distress, SpO2 < 90% at ICU admission, and/or labored breathing. Exclusion criteria were admission for another cause, hypercapnia (defined by a PaCO2 > 50 mmHg), and invasive mechanical ventilation before ICU admission. Secondly, we used the HIGH trial [3], a RCT which enrolled 776 immunocompromised patients with hypoxemic ARF in 31 ICUs, in order to compare HNFC to standard oxygen at day-28 mortality. In both studies, each participating center has a senior intensivist and a senior hematologist in site available 24/7 and sharing decisions of ICU admission [17]. Note that 14 centers were common in both the TRIAL-OH cohort and the HIGH trial.

The appropriate ethics committees approved each study.

### Variables and risk adjustment

The primary outcome was the need for intubation through ICU stay as a dichotomous variable. In the TRIAL-OH study, the decision to perform endotracheal intubation was left to clinicians in charge at each ICU. In the HIGH trial, the decision to perform endotracheal intubation was based on predefined criteria, in agreement with current guidelines, including the therapeutic response and the clinical status (SpO2, respiratory rate, signs of respiratory distress, and bronchial secretion volume) [3]. Individual data reported in tables were collected prospectively. ICU-level data (i.e., center characteristics) were also used in the analysis. In the TRIAL-OH cohort, these data were assessed as part of data collection. In the HIGH trial, they were assessed with a secondary survey from all the participant centers.

### Statistical analysis

Continuous variables are described as median and interquartile range (IQR), and categorical variables are summarized by counts (percent).

Patient characteristics at admission were examined at the patient level in both studies, and the need for intubation was first assessed in each center without adjusting on potential confounding factors, which is referred as the “crude need for intubation.” Hierarchical logistic regression models were used to examine the variability on the outcome between ICUs and the association between ICU characteristics and the outcome, adjusting for patient characteristics [18]. To do this, we used the mixed-effect model with a normally distributed random effect for ICU (random intercept). Exchangeability was assumed across all providers [5]. In practice, the effect of a given ICU was modeled through its own regression coefficient, which compares to the crude average need for intubation across all centers [5, 6]. This allows us to model between ICU heterogeneity in the average intubation risk. These models provide an estimation of heterogeneity in the form of the variance of the random effects, where the closer the variance is to zero, the smaller the center effect is. Because it could be hard to interpret, we computed the median odds ratio (MOR) to better understand the importance of the center effect on the mean intubation risk, that is on the same scale as traditional prognostic factors [6, 7, 18, 19]. Briefly, MOR corresponds to the median of all ORs that can be computed between two patients with the same characteristics, but randomly chosen from two different centers, namely in a higher risk center and in a lower risk one [6, 18]. MOR quantifies the differences between ICUs. If the MOR equals 1, then there are no differences in intubation risk between ICUs.

First, the model was built without any adjustment (“empty model”), which allows us to investigate a potential center effect. This unadjusted model contained only ICU-specific random intercepts. Then, we provided adjustment on predefined individual covariates as fixed effects, without ICU characteristics or interventions. These covariates were specified a priori as potential confounders and included age, comorbidities assessed by the Charlson comorbidity index, type of immunosuppression (in three classes, malignant hemopathy, solid cancer, and others), previous allogeneic stem cell transplantation, performance status > 2 (bedridden or dependent), severity of illness with SOFA score without respiratory item, Pa0_2_/FiO_2_ ratio in four categories (> 300, 200–300, 100–200, ≤ 100 mmHg, with > 300 mmHg as reference), respiratory rate > 30 /min, and ARF diagnosis. This model was used to estimate adjusted ICU random effects, because we were interested in studying heterogeneity in outcomes. ICU were then ranked by their estimated random effect on intubation risk (adjusted for patient characteristics only) and classified into quartiles. For descriptive purposes, patient and ICU characteristics were compared across quartiles of the risk-adjusted intubation rate, using the Cochran–Mantel–Haenszel row mean score test and nonzero correlation test for testing the difference for categorical and continuous variables, respectively. To illustrate the magnitude of the effect of center on intubation risk, we performed conditional standardization of the regression results for a given patient with median and modal values for the covariates in the patient-level adjusted model. Last, hospital- and ICU-level covariates (center volume, annual volume of ID in ICU, annual rate of IMV patients defined by the number of patients with IMV/number of admission, academic status of hospital) were entered in the model as fixed effects and reported in terms of odds ratios, in order to try to explain the discrepancies between center. Because the time since respiratory symptoms onset and ICU admission could reflect local practices, it was considered as an ICU-level characteristic in the analysis.

To test for the significance of the center effects (empty model and model adjusted on patient and center characteristics), we used permutations test, a recommended approach to test for random effect [20–23]. More details about the methods used are given in the Additional file 1.

These methods were applied in both the observational cohort and the RCT, separately.

### Sensitivity analyses

Primary analyses were performed on the complete cases, assuming missing completely at random covariates. Then, sensitivity analyses for such assumptions were performed, based on multiple imputation with chained equation [24]. We performed exploratory subset analyses, restricting ourselves to patients with full code status, in patients with malignant diseases in the HIGH trial and after exclusion of ICUs with extreme size (ICUs > 20 beds or ICUs< 8 beds). In the HIGH trial, we also investigated the center heterogeneity in the prognostic effect of the oxygenation strategy which was randomly assigned (HFNC or standard oxygen). This was not possible in the observational cohort due to allocation bias. To do this, we introduced two random effects in the patient-level adjusted model for each center: a random effect on the mean intubation (random intercept) risk as previously described and a random effect on the effect of the oxygenation strategy (with standard oxygen as reference) on intubation risk random slope. This allows us to model centers’ variability not only on the average intubation risk but also on the effect of the oxygenation strategy on the need for intubation. We then applied permutation test to investigate for significance of center effect [20, 22].

All reported *p* values are two-sided; *p* < .05 was considered statistically significant. All analyses were performed using R version 3.1.0 (R Foundation for Statistical Computing [http://www.R-project.org/]).

## Results

### Patients

Overall, 703 patients (age 61.0 years [51.0–71.0], 61.0% male) were included in the observational cohort and 776 patients (age 64.0 years [56.0–71.0], 66.2% male) from the HIGH trial (Additional file 1: Figure S1). Table 1 reports the main characteristics at ICU admission and the respiratory parameters at baseline. As shown, the main cause of immunosuppression was hematological malignancies in both studies (94% in the observational cohort and 45% in the RCT). Most of the patients were admitted from ward with a good performance status (i.e., 0–2) over the 3 months preceding ICU admission (78.8% and 63.7% respectively). Sequential Organ Failure Assessment (SOFA) score at admission was 6 [3, 4, 8–12, 25] and 6 [4, 8–10, 25] the in cohort and trial respectively. In both studies, the main diagnosis of ARF was bacterial infection (269 (38.2%) in the Trial-OH cohort, 355 (45.7%) in the HIGH trial) followed by opportunistic germ infections (89 (12.6%) and 93 (12.0%)).

**Table 1:** Characteristics of patients at ICU admission

Demographic and clinical data	Trial-OH cohort (*n* = 703)	High randomized controlled trial (*n* = 776)
Characteristics of the patients		
Demographics		
Age, median [IQR], years	61 [51.0–71.0]	64.0 [56.0–71.0]
Male sex, *n* (%)	429 (61.0)	517 (66.2)
BMI, kg/m^2^	24.6 [21.6–27.7]	24.9 [22.3–28.1]
Source of admission		
ER or ambulance	298 (42.4)	327 (42)
Night or weekend admissions	434 (61.7)	
Comorbidities		
Respiratory	199 (28.3)	242 (31.2)
Heart failure	98 (14)	50 (6.4)
Kidney disease	64 (9)	97 (12.5)
Charlson score	4 [3.0–6.0]	5.0 [3.0–7.0]
Underlying conditions		
Hematologic malignancies	665 (94.6)	348 (45)
Solid tumors	38 (5.4)	265 (34)
Immunosuppressive drugs	–	268 (35)
Remission of malignancy	293 (41.7)	141 (18.2)
Autologous stem cell transplantation	104 (14.8)	48 (6.2)
Allogeneic stem cell transplantation	113 (16.1)	61 (7.9)
Poor performance status (> 2)	149 (21.2)	282 (36.3)
Clinical parameters at baseline		
Respiratory rate, breaths/min	32 [26.0–38.0]	28.0 [23.0–33.0]
Oxygen flow, l/min	6 [3.0–10.0]	11.0 [6.0–15.0]
Glasgow coma score	15 [15.0–15.0]	15.0 [15.0–15.0]
Neutropenia	203 (28.9)	136 (17.5)
Number of quadrants on chest X-ray	2.0 [1.0–4.0]	2.0 [2.0–4.0]
Arterial blood gas at baseline		
pH, units	7.40 [7.31–7.46]	7.43 [7.39–7.47]
PaCO_2_, mmHg	37.0 [31.0–44.0]	35.0 [30.5–39.0]
PaO_2_/FiO_2_ ratio, mmHg	161.4 [113.0–226.4]	132.0 [93.0–176.0]
Severity of PaO_2_/FiO_2_ ratio at baseline		
PaO_2_/FiO_2_ > 300 mmHg	100 (14.2)	38 (4.9)
PaO_2_/FiO_2_ 200–300 mmHg	125 (17.8)	104 (13.4)
PaO_2_/FiO_2_ 100–200 mmHg	340 (48.4)	351 (45.2)
PaO_2_/FiO_2_ ≤ 100 mmHg	138 (19.6)	231 (29.8)
Organ dysfunction at day 1		
SOFA score without respiratory item	5.00 [3.0–8.0]	3.00 [3.0–4.0]
Use of vasopressor	225 (32.0)	117 (15)
ARF etiology		
Bacterial infection	269 (38.2)	355 (45.7)
Opportunist germs infection	89 (12.6)	93 (12.0)
Disease-related infiltrates/drug-related toxicity	73 (10.4)	92 (11.9)
Cardiogenic pulmonary edema	58 (8.2)	8 (1.0)
Undetermined	100 (14.2)	176 (22.7)
Others	104 (14.8)	103 (13.3)
NA	10 (1.4)	2 (0.3)
Outcome		
Invasive mechanical ventilation	398 (57)	320 (41.5)
ICU mortality	228 (32.4)	245 (31.6)
Hospital mortality	309 (44.0)	322 (41.5)
ICU length of stay	6.0 [3.0–12.0]	7.0 [4.0–13.5]
Hospital length of stay, days	15.0 [7.0–29.0]	26.0 [15.0–42.0]

Values are given in *N* (%) or median [IQR]

*Abbreviations*: *IQR* interquartile range, *ICU* intensive care unit, *SOFA* Sequential Organ Failure Assessment, *BMI* body mass index, mo months

ICU and hospital mortality rates were 32.4% (228 deaths) and 44% (309 deaths) in the cohort and 31.6% (245 deaths) and 41.5% (322 deaths) in the trial, respectively.

### Center effect on endotracheal intubation risk

#### Patient-level analysis

**Fig. 1:**
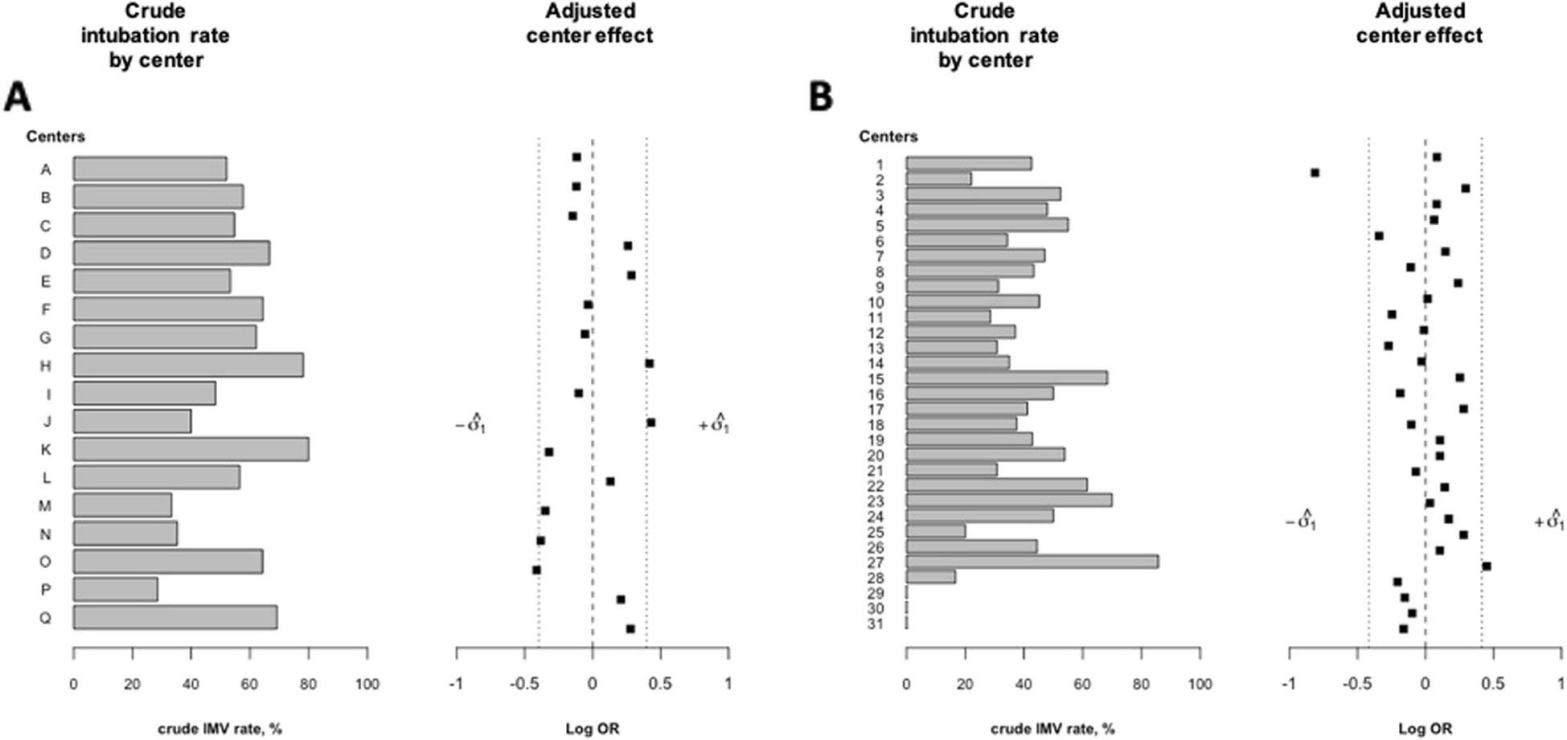
Crude intubation rate by center (left) and distribution of center effects on intubation rate (right) adjusted on individual confounders. Centers are sorted by study size. Black squares represent adjusted center effects on the mean intubation risk as odds ratio (OR) (comparison of each center to a theoretical average reference center with OR = 1). **a** The TRIAL-OH cohort. **b** The HIGH trial. *Abbreviations*: IMV invasive mechanical ventilation, OR odds ratio

Center size distribution is summarized in Additional file 1: Table S1 and S2. In the TRIAL-OH cohort, the crude intubation rate was 57% (398 patients, median delay of 0 day [0–1]) and ranged from 29 to 80% across ICUs. In the HIGH trial, the crude intubation rate was 41.5% (320 patients, 1 day [0–2]) and ranged from 0 to 86% in the different participating centers (Fig. 1). Of note, 45% (181 patients) of intubation procedure were performed during nighttime or weekend in the TRIAL-OH cohort and 56% (181 patients) in the HIGH trial.

In both studies, we found a significant variation in the crude intubation risk across ICUs (TRIAL-OH cohort and HIGH trial, *p* < 0.01). Median OR for baseline intubation risk were 1.38 [1.24–1.56] and 1.37 [1.26–1.50], respectively.

We then performed multivariable analyses to adjust on individual potential confounders for endotracheal intubation. As shown in Table 2, in both studies, there was a significant center effect on intubation risk after adjustment on potential individual confounders (TRIAL-OH cohort, *p* value 0.013; HIGH trial, *p* value 0.004). The magnitude of the center effect is depicted in Fig. 1 a and b. As shown, there was a significant variation of intubation risk among the different participant ICUs. Median OR for center effect on the mean intubation risk were 1.48 [1.30–1.72] (TRIAL-OH cohort) and 1.51 [1.36–1.68] (HIGH trial). In other words, the center effect persisted after adjustment for individual patient characteristics.

**Table 2:** Results of the multivariable mixed regression model with center effect on subsequent risk for endotracheal intubation

	Trial-OH cohort	High randomized controlled trial
No. of intubation/no. of observation	398/703	320/776
Center effect*		
Estimated true inter-hospital variance**	0.156	0.186
Median odds ratio***	1.48 [1.30–1.72]	1.51 [1.36–1.68]
Predicted probability of intubation, mean (min-max) across centers	0.56 (0.46–0.70)	0.41 (0.28–0.55)
*p* value for center effect	0.013	0.004

*Results were adjusted on age, Charlson comorbidity index, type of immunosuppression, allogeneic stem cell transplantation, sex, performance status > 2, diagnosis of acute respiratory failure, Pa0_2_/FiO_2_ ratio in four categories (> 300, 200–300, 100–200, ≤100 mmHg, with > 300 mmHg as reference), respiratory rate > 30/min, SOFA score without respiratory item

**Intercept variance

***The median odds ratio (MOR) is defined as the median value of the odds ratio between the hospital at highest risk and the hospital at lowest risk for two randomly chosen hospitals

**Fig. 2:**
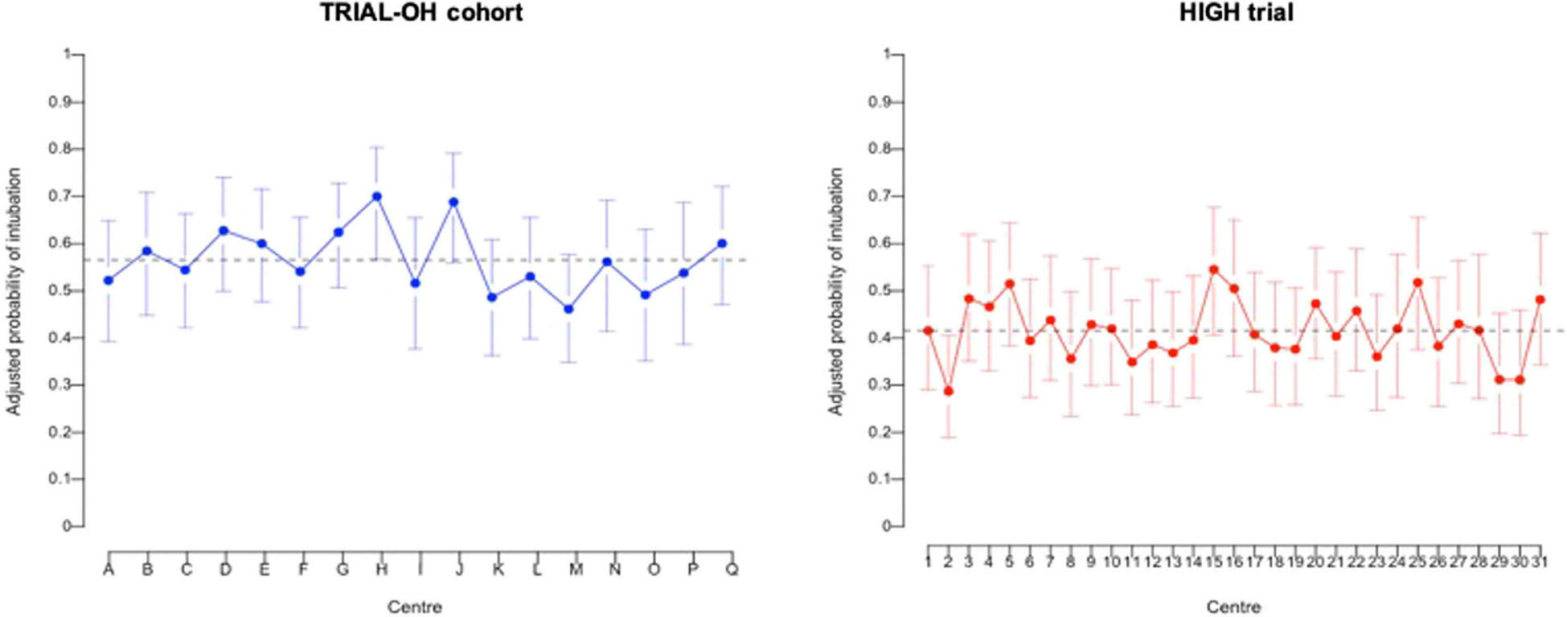
Absolute risk of intubation according to center calculated with the use of conditional standardization of the regression results. Adjusted conditional risk of intubation (with 95% confidence intervals) shows the predicted risk of intubation for a patient at approximately the 50th percentile of risk in each center—for example, in the HIGH trial, a 64-year-old patient with hematologic malignancy, a Charlson score at 5, performance status < 2, and a SOFA score without respiratory item at 3 who was admitted for bacterial pneumonia with a PaO2/Fi02 between 100 and 200 and a respiratory rate at 30/min

Also, given exact same baseline prognostic factors, the predicted intubation risk of two patients selected in different ICU ranged from 46 to 70% in the TRIAL-OH cohort and from 28 to 55% in the HIGH trial (Table 2 and Fig. 2).

#### ICU-level analysis

Differences in patient and hospital characteristics and outcomes by hospital-adjusted intubation rate quartiles are illustrated in Additional file 1: Tables S3-S6 and Figure S2. As expected, baseline patient characteristics were well balanced between the groups after case-mix adjustment. Nonetheless, in both TRIAL-OH cohort and HIGH trial, patients treated at ICU in the higher quartiles (Q3 and Q4) of intubation risk were more likely to have a good performance status. At the ICU level, the only difference was the annual rate of patients whom required invasive mechanical ventilation. We then adjusted the analysis on ICU-level characteristics. Table 3 presents intubation risk as a function of ICU characteristics. As shown, the annual IMV rates and the time from respiratory symptoms onset to ICU admission were both associated with a greater risk for intubation. Adjustment on these two factors significantly reduces the variability in the intubation risk which was no longer significant (Additional file 1: Figure S3).

**Table 3:** Intensive care unit characteristics associated with intubation risk

Hospital characteristics	Trial-OH cohort		High randomized controlled trial	
	OR* [95% CI]	*p* value	OR* [95% CI]	*p* value
Teaching hospital	1.04 [0.55; 1.96]	0.89	0.84 [0.46;1.52]	0.56
No. of hospital beds, per 100 beds	0.99 [0.94; 1.04]	0.77	0.94 [0.87;1.01]	0.11
No. of ICU beds, per 10 beds	0.96 [0.83; 1.12]	0.67	0.93 [0.66;1.31]	0.70
Annual volume of ID patients	0.95 [0.75; 1.22]	0.72	1.00 [0.81;1.23]	0.99
Annual IMV rate	1.26 [1.04; 1.53]	0.01	1.28 [1.02;1.62]	0.03
Time from respiratory symptoms to ICU admission, by day	1.08 [1.02; 1.15]	0.005	1.10 [1.02; 1.87]	0.02

*Results were adjusted on age, Charlson comorbidity index, type of immunosuppression, allogeneic stem cell transplantation, sex, performance status> 2, diagnosis of acute respiratory failure, Pa0_2_/FiO_2_ ratio in four categories (> 300, 200–300, 100–200, ≤100 mmHg, with > 300 mmHg as reference), respiratory rate > 30 /min, SOFA score without respiratory item

*Abbreviations*: *ICU* intensive care unit, *ID* immunocompromised patients, *IMV* invasive mechanical ventilation

#### Sensitivity analyses

In the HIGH trial, we investigated the effect of center not only on the average intubation risk but also on the intervention arm effect (HFNC use). As previously described, a global test found a significant center effect (*p* value 0.037) on the mean risk for intubation. However, we did not find any significant between-center heterogeneity of the effect of HFNC use on subsequent intubation risk (*p* value 0.188, Additional file 1: Figure S4). This suggests that the effect of treatment on survival did not vary across centers, but that a significant heterogeneity in the distribution of outcomes was reported.

To explore how stable were these findings, we repeated the analysis under varying assumptions. Excluding ICUs with extreme sizes did not change our results (*p* value for center effect 0.013 (TRIAL-OH cohort) and 0.003 (HIGH trial). In the HIGH trial, our results were not affected by the exclusion of patients with “do-not-intubate order” through ICU course (34 patients excluded, *p* value for center effect 0.005) or when we restricted our analysis to onco-hematological patients only (613 patients, *p* value for center effect 0.028). Finally, analyses performed after multiple imputation of missing data led to the same results (TRIAL-OH cohort, *p* value for center effect 0.012, median OR 1.41 [1.26–1.61]; HIGH trial, *p* value for center effect < 0.01, median OR 1.36 [1.25–1.47]).

## Discussion

This study was the first to explore variability across centers of the risk for invasive mechanical ventilation in critically ill immunocompromised patients with ARF. The significant variability in intubation rates applies to both a large observational cohort and a large randomized controlled trial. Moreover, the significant variability persisted after adjustment on potential individual confounders for IMV. Furthermore, the magnitude of the center effect, summarized herein with the median OR, was quite large. Last, we identified two ICU-level characteristics which could partly explain the observed discrepancy.

These findings suggesting between-center heterogeneity in intubation risk raise several concerns. In immunocompromised patients, ARF is the leading cause for ICU admission, the need for IMV being near 50% and mortality rates reaching up to 70% [26–28]. Also, strategies to improve oxygenation and avoid invasive mechanical ventilation have received a great attention over the last two decades [29, 30]. It is generally admitted that an ideal trial endpoint should be clinically relevant, accepted in medical practice, and sensitive and specific to detect the anticipated effect of the treatment [31]. In the ICU setting, mortality has long been a major criterion. However, numerous RCTs were deemed negative because of the inappropriateness of the primary endpoint [32]. As a consequence, the need for endotracheal intubation could appear as a better target in ARF and has become the commonest primary endpoint of more recent RCTs [8, 10, 25]. However, in this study, we found that the adjusted intubation risk ranged from 46 to 70% in an observational study and from 28 to 55% in a RCT. Hence, evidences for a significant variation in intubation rate across different ICUs could challenge the validity of such outcome.

Different reasons could be argued to explain these discrepancies: First, organizational practices and local admission policies. In this study, we found that intubation risk increased with the annual ratio of patient with IMV in a given center, and the time from respiratory symptom onset to ICU admission. One could suppose that these two factors could reflect local practices for ICU admission and IMV initiation. Second, physician experience as well as patient conditions could influence the intubation decision [33]. This point is hard to capture in statistical analysis, but in our study, we found a significant variation in intubation rate despite predefined intubation criteria. Moreover, most of intubation procedures have been made during out of hour period, supporting the importance of workload and personal practice factors. In a study which focused on IMV initiation in septic shock patients, de Montmollin et al. found a good agreement in a panel of intensivist for hypoxemia and respiratory rate as criteria for the need of intubation [34]. However, in our study, discrepancies persisted after adjustment on these factors. These two points encourage the need for define consensual intubation criteria, as it appears to date as an important outcome. Last, patient severity is an important factor and could vary across centers, but in the present study, a significant center effect on intubation risk persisted even after adjustment on individual confounders.

These findings emphasize the need for several considerations. First, mortality is probably an unrealistic outcome in an era of numerous negative trials and endotracheal intubation appears to suffer for large center-related source of variation due to case-mix heterogeneity and more importantly local practices. Non-mortal outcome such as patients reported outcome or variation of severity parameters (delta-SOFA or oxygenation parameters) could be taken into consideration for future trials [32]. Then, in observational studies and maybe RCT, it could be of importance to take into account between center variation in analysis. In this way, the random-effect logistic model could be of interest [35, 36]. Finally, the large variation in endotracheal rate across centers despite adjustment on individual characteristics and patient severity could suggest variation in the decision of endotracheal intubation. As it remains an important outcome in clinical studies, there is maybe the need for a consensual definition of IMV implementation.

This study has several limitations. First, it was a post hoc analysis and we cannot rule the inherent limitations of these methods. However, using adequate statistical methods, we found significant center variations in intubation risk, identified potential explicative factors, and assessed its importance. Second, although we attempted to address ICU variation by adjusting for patient-level clinical factors known to be related with intubation risk, but the possibility of confounding by unmeasured covariates remains. Third, we used data from two studies performed in specialized centers. This could limit the generalizability of the results. Last, we cannot exclude that some of the patients could have been intubated for non-respiratory reasons (i.e., coma, severe shock, or copious tracheal secretions). However, our record of non-respiratory SOFA and the adjustments that were made in the analyses actually takes into account this issue.

## Conclusion

Invasive mechanical ventilation has become an important endpoint in immunocompromised patients with acute respiratory failure. However, we found significant variation in intubation risk across ICU in both an observational cohort and a randomized controlled trial. Our results highlight the need to take into account center effect in analysis because it could be an important confounder. Reasons for heterogeneity are various (case-mix differences, center practices). This gives opportunities to future improvement in care management and study design.

## Additional file

Additional file 1: Further explication on statistical methods. **Table S1.** Center size distribution in the Trial-OH cohort and crude invasive mechanical ventilation rate by center. **Table S2.** Center size distribution in the HIGH trial and crude invasive mechanical ventilation rate by center. **Table S3**. Patient characteristics by quartiles of ICU adjusted rates of invasive mechanical ventilation- TRIALOH-Cohort (*n* = 703). **Table S4.** Center characteristics by quartiles of ICU adjusted rates of invasive mechanical ventilation-TRIAL-OH cohort (*n* = 17). **Table S5**. Patient characteristics by quartiles of ICU adjusted rates of invasive mechanical ventilation-HIGH-trial (*n* = 776). **Table S6**. Center characteristics by quartiles of ICU adjusted rates of invasive mechanical ventilation-HIGH Study (*n* = 31). **Figure S1:** Flow chart of the study. **Figure S2.** Adjusted probability of intubation. **Figure S3.** Ranking of ICUs by center effect on the mean intubation risk with and without adjustment on annual invasive mechanical ventilation rate and time from respiratory symptoms to ICU admission. **Figure S4.** Center effect on the effect of oxygenation strategy (High Flow Nasal cannula) on Intubation risk in the High Trial. (DOCX 13509 kb)

## Data Availability

The datasets used and/or analyzed during the current study are available from the corresponding author on reasonable request.

## Abbreviations

ARF: Acute respiratory failure
HFNC: High-flow nasal cannula
ICU: Intensive care unit
IMV: Invasive mechanical ventilation
IQR: Interquartile range
MOR: Median odds ratio
NIV: Noninvasive ventilation
PS: Performance status
RCT: Randomized controlled trial
SOFA: Sequential Organ Failure Assessment
