# Supplementary material for: Center effect in intubation risk in critically ill immunocompromised patients with acute hypoxemic respiratory failure
Source: Crit Care. 2019 Sep 6;23:306. doi: 10.1186/s13054-019-2590-7 (PMC6731598; doi:10.1186/s13054-019-2590-7)
Supplement: Supplementary file 1 — Further explication on statistical methods. Table S1. Center size distribution in the Trial-OH cohort and crude invasive mechanical ventilation rate by center. Table S2. Center size distribution in the HIGH trial and crude invasive mechanical ventilation rate by center. Table S3. Patient characteristics by quartiles of ICU adjusted rates of invasive mechanical ventilation- TRIALOH-Cohort (n = 703). Table S4. Center characteristics by quartiles of ICU adjusted rates of invasive mechanical ventilation-TRIAL-OH cohort (n = 17). Table S5. Patient characteristics by quartiles of ICU adjusted rates of invasive mechanical ventilation-HIGH-trial (n = 776). Table S6. Center characteristics by quartiles of ICU adjusted rates of invasive mechanical ventilation-HIGH Study (n = 31). Figure S1: Flow chart of the study. Figure S2. Adjusted probability of intubation. Figure S3. Ranking of ICUs by center effect on the mean intubation risk with and without adjustment on annual invasive mechanical ventilation rate and time from respiratory symptoms to ICU admission. Figure S4. Center effect on the effect of oxygenation strategy (High Flow Nasal cannula) on Intubation risk in the High Trial. (DOCX 13509 kb) [file 13054_2019_2590_MOESM1_ESM.docx]

**Additional file 1**

**Center effect in intubation risk in critically-ill immunocompromised patients with acute hypoxemic respiratory failure.**

**Authors:**

Guillaume Dumas, MD; Alexandre Demoule, MD, PhD; Djamel Mokart, MD, PhD; Virginie Lemiale, MD; Saad Nseir, MD, PhD; Laurent Argaud, MD, PhD; Frédéric Pène, MD, PhD; Loay Kontar, MD; Fabrice Bruneel, MD; Kada Klouche, MD, PhD; François Barbier, MD, PhD; Jean Reignier, MD, PhD; Annabelle Stoclin, MD; Guillaume Louis, MD; Jean-Michel Constantin, MD, PhD; Florent Wallet, MD; Achille Kouatchet, MD; Vincent Peigne, MD; Pierre Perez, MD; Christophe Girault, MD; Samir Jaber, MD, PhD; Yves Cohen, MD; Martine Nyunga, MD; Nicolas Terzi, MD, PhD; Lila Bouadma, MD, PhD; Christine Lebert, MD; Alexandre Lautrette, MD, PhD; Naike Bigé, MD, PhD; Jean-Herlé Raphalen, MD; Laurent Papazian, MD, PhD; Dominique Benoit, MD; Michael Darmon, MD, PhD; Sylvie Chevret, MD, PhD; Elie Azoulay, MD, PhD.

**Corresponding author:**

Professor Elie Azoulay,

Medical Intensive Care Unit, Hôpital Saint-Louis,

ECSTRRA team, Biostatistics and clinical epidemiology, UMR 1153 (center of epidemiology and biostatistic Sorbonne Paris Cité, CRESS), INSERM, Paris Diderot University

Mailing address: Medical Intensive Care Unit, Hôpital Saint-Louis, 1 avenue Claude Vellefaux, 75010 Paris France

E-mail: elie.azoulay@aphp.fr

1. **Flow chart**

**Figure S-1:** Flow chart of the study

1011

patients with onco-hematologic malignancies admitted in ICU

**TRIAL-OH**

**cohort**

703 patients with acute respiratory failure

308 patients without acute respiratory failure

17 centers

invasive mechanical Ventilation : 398 (57 %)

In Hospital mortality: 309 (44 %)

**HIGH**

**randomized control trial**

776 immunocompromised patients in ICU with acute respiratory failure

31 centers

invasive mechanical Ventilation : 320 (41 %)

In Hospital mortality: 322 (42 %)

**Statistical appendix.**

1. **Center effect model**

Hierarchical regression models were used to examine the variability on outcome between intensive care units (ICU) and the association between ICU characteristics and outcome, adjusting for patient characteristics.

We used mixed-effect logistic regression model with center as random variable and patients and hospital variables as fixed effect. In all model we assume the exchangeability of centers. In practice, the effect of a given center j was modeled through its own coefficient bi_j_ which compares to the crude average intubation risk across all centers.

Also, in absence adjustment on patients-level characteristics, the model of the hospital intubation risk for a patient i, in center j is given by:

$$\mathrm{Logit}\left( \mathrm{pij} \right)=\log\left( \frac{\mathrm{pij}}{1-pij} \right)=I+bj$$

Where:

I corresponds to the crude average ICU intubation risk expressed on logistic scale

bj (random intercept), is assumed to be normally distributed with a mean 0 and a variance Vj

(that is the variance around I).

Hence, the probability of intubation for a patient i in center j, depends on I and bj and could be rewritten as follow:

$$pi=\frac{\exp\left( I+bj \right)}{1+exp(I+bj)}$$

In the model with adjustment on patients-level characteristics (X1, X2,X3), the probability of intubation depends on center and individual covariates :

$$\mathrm{Logit}\left( \mathrm{pij} \right)=I+ \beta1X1+\beta2X2+\beta3X3+bj$$

Where, β1, β2, β3 are the regression coefficients for the individual covariates (fixed effect) and bj the random intercept on the mean ICU intubation risk.

These models provide estimates of the between-center heterogeneity in the form of the variances of the random effects Vj. In the absence of center effect, these variances are equal to zero. Also, the higher is the variance, the higher is the center effect.

1. **Median odds ratio**

The Median odds ratio (MOR) is used to translate center variance (i.e. heterogeneity) in odds ratio scale, allowing a more intuitive interpretation. It is defined as the median value of the odds ratio which can be computed between two patients with the same characteristics but from two different ICU. It’s always computed from the patient in the higher risker center to the patient in the lower-risk center and thus, MOR is greater than one. Interestingly, it is statistically independent of the prevalence of the event of interest (here the intubation prevalence).

If the MOR was equal to one, there would be no differences between ICU in the probability of intubation[1, 2].

1. **Permutation test**

Permutation tests is a procedure which have been recommended for random effects testing. It is more parsimonious and has better properties than likelihood ratio or Wald tests where center is considered as fixed effect[3, 4]. Permutation tests have been validated in previous studies for the test of both a single and multiple intercept random effect in generalized linear mixed models[5, 6]. The principle is to test whether the random effects variance component is zero. In our situation, the objective is to test if the random effects variance component for center is zero. In practice, we would randomly permute the center that the patient was assigned to, while keeping the number of patients assigned to a given center the same as in the original sample.

The null hypothesis is no effect center. Under H0, the outcome is independent of centers and therefore, the center indices is exchangeable. The procedure of permutation following the different steps above :

- First, the statistic is compute on the original dataset
- Then the center indices is randomly permute a large number of times (here 1000 times)
- On each permuted sample, we compute the statistic
- An approximate distribution of the statistic is obtain under H0 by a large number of permutation
- The empirical p-value being the proportion of permutation samples with a statistic greater than or equal to those observed on the original sample
- Reject of H0 if the prespecified risk α is greater than the empirical p-value.

In our case, we used a test statistic proposed in linear mixed model and which relies on the estimates of the variance components of the random effects [5, 7, 8].

**Illustration of permutation procedure**


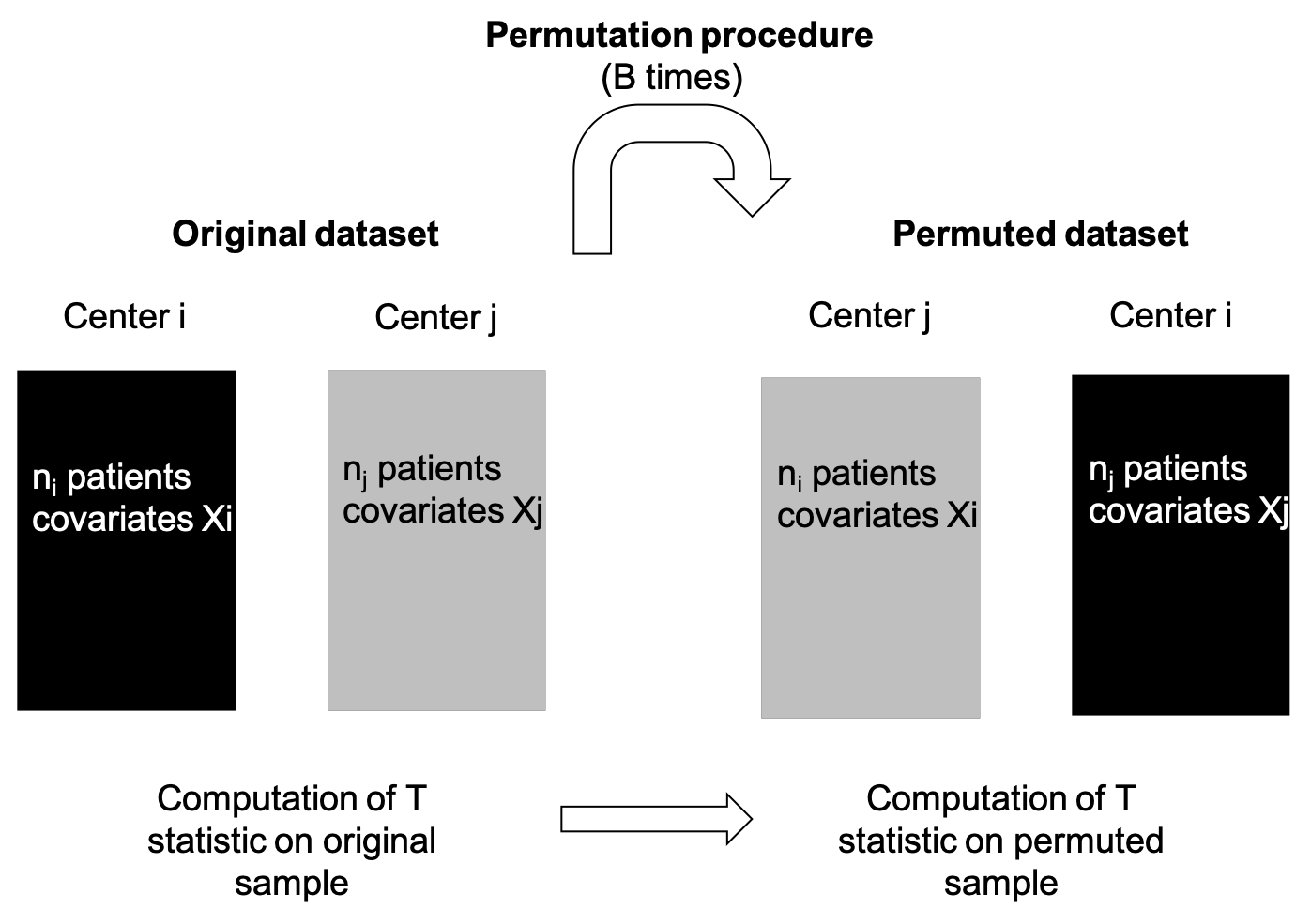


The original dataset is composed of two center i and j with respectively n_i_ and n_j_ patients.

B permutation are computed (1000 times) on the original dataset. With a sufficient number of permutation under the null hypothesis, we obtain the approximate distribution of T and a p-value could be calculated.

1. **Centers characteristics**

**Table S-1.** Center size distribution in the Trial-OH cohort and crude invasive mechanical ventilation rate by center

| Variable | No. (%) | Endotracheal rate No. (%) |
| --- | --- | --- |
| Total | 703 | 398 (57) |
| Centers |  |  |
| A | 144 (20) | 75 (52) |
| B | 104 (15) | 60 (58) |
| C | 64 (9) | 35 (55) |
| D | 54 (8) | 36 (67) |
| E | 45 (6) | 29 (64) |
| F | 45 (6) | 24 (53) |
| G | 37 (5) | 23 (62) |
| H | 32 (5) | 25 (78) |
| I | 29 (4) | 14 (48) |
| J | 25 (4) | 20 (80) |
| K | 25 (4) | 10 (40) |
| L | 23 (3) | 13 (57) |
| M | 18 (3) | 6 (33) |
| N | 17 (2) | 6 (35) |
| O | 14 (2) | 4 (29) |
| P | 14 (2) | 9 (64) |
| Q | 13 (2) | 9 (69) |

**Table S-2.** Center size distribution in the HIGH trial and crude invasive mechanical ventilation rate by center

| Variable | No. (%) | Endotracheal rate No. (%) |
| --- | --- | --- |
| Total | 776 | 320 (41.5) |
| Centers |  |  |
| 1 | 101 (13.1) | 43 (43) |
| 2 | 77 (10) | 17 (22) |
| 3 | 61 (7.9) | 32 (52) |
| 4 | 48 (6.2) | 23 (48) |
| 5 | 40 (5.2) | 22 (55) |
| 6 | 35 (4.5) | 12 (35) |
| 7 | 34 (4.4) | 16 (34) |
| 8 | 32 (4.1) | 10 (32) |
| 9 | 30 (3.9) | 13 (30) |
| 10 | 31 (4) | 14 (45) |
| 11 | 28 (3.6) | 8 (28) |
| 12 | 27 (3.5) | 10 (27) |
| 13 | 26 (3.4) | 8 (26) |
| 14 | 20 (2.6) | 7 (35) |
| 15 | 19 (2.5) | 13 (68) |
| 16 | 18 (2.3) | 9 (50) |
| 17 | 17 (2.2) | 7 (35) |
| 18 | 16 (2.1) | 6 (38) |
| 19 | 14 (1.8) | 6 (43) |
| 20 | 13 (1.7) | 8 (62) |
| 21 | 13 (1.7) | 4 (31) |
| 22 | 13 (1.7) | 7 (54) |
| 23 | 10 (1.3) | 2 (20) |
| 24 | 10 (1.3) | 5 (50) |
| 25 | 10 (1.3) | 7 (70) |
| 26 | 9 (1.2) | 4 (44) |
| 27 | 7 (0.9) | 6 (86) |
| 28 | 6 (0.8) | 1 (17) |
| 29 | 3 (0.4) | 0 (0) |
| 30 | 2 (0.3) | 0 (0) |
| 31 | 2 (0.3) | 0 (0) |

1. **Distribution of adjusted invasive mechanical ventilation probability**

**Figure S-2.** Adjusted probability of intubation


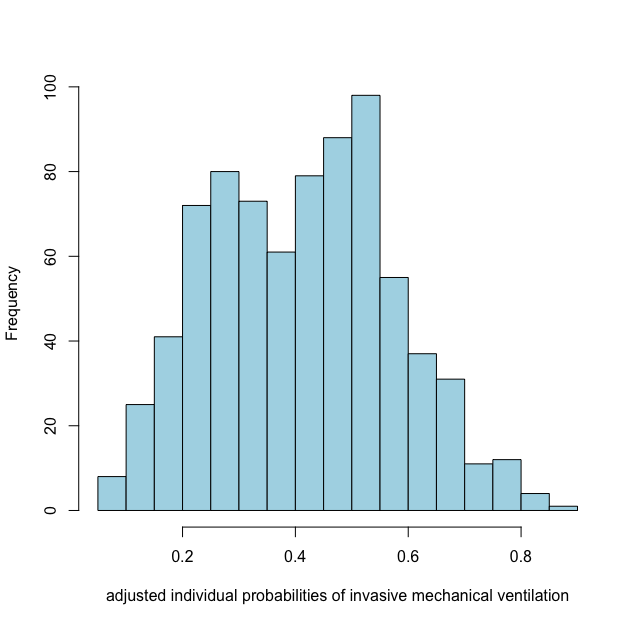


**HIGH trial**

**TRIAL-OH Cohort**


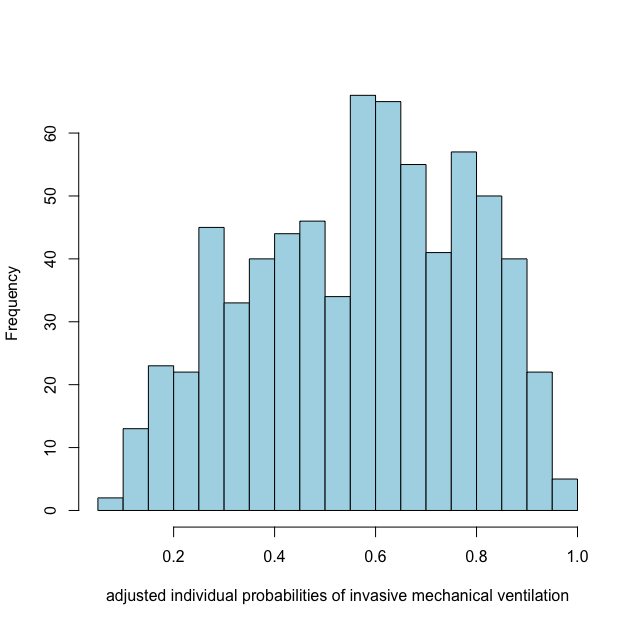


1. **Comparison of patients and centers characteristics according to quartiles of risk adjusted for invasive mechanical ventilation**

**Table S-3**. Patient characteristics by quartiles of ICU adjusted rates of invasive mechanical ventilation- TRIALOH-Cohort (n=703)

|  | **Quartile 1**  **(Lowest)** | **Quartile 2** | **Quartile 3** | **Quartile 4 (Highest)** | **p-value** |
| --- | --- | --- | --- | --- | --- |
| **Intubation rate, %** | 44 | 55 | 60 | 72 | <0.001 |
| **Characteristics of the patients** |  |  |  |  |  |
| Age, median [IQR], year | 64.0 [57.2, 74.0] | 60.0[47.2, 68.0] | 59.0 [49.7, 70.0] | 65.0 [55.0, 75.5] | 0.49 |
| Male sex, n (%) | 76 (55.1) | 188 (59.9) | 84 (61.8) | 81 (70.4) | 0.01 |
| Charlson score | 4.0 [3.0, 6.0] | 4.0 [2.0, 5.0] | 4.0 [3.0, 5.5] | 5.0 [3.0, 6.5] | 0.42 |
| Respiratory comorbidity | 47 (34.1) | 83 (26.4) | 35 (25.7) | 34 (29.6) | 0.46 |
| Hematologic malignancy | 130 (94.2) | 298 (94.9) | 132 (97.1) | 105 (91.3) | 0.52 |
| Allogeneic stem cell transplantation | 18 (13.0) | 58 (18.5) | 28 (20.6) | 9 ( 7.8) | 0.35 |
| Poor performance status (>2) | 28 (20.3) | 86 (27.4) | 22 (16.2) | 13 (11.3) | 0.01 |
| Time from respiratory symptoms to ICU admission, day | 0.50 [0.0, 2.0] | 1.0 [0.0, 3.0] | 1.0 [0.0, 4.0] | 0.0 [0.0, 1.50] |  |
| **Clinical parameters at baseline** |  |  |  |  |  |
| Neutropenia | 38 (27.5) | 81 (25.8) | 50 (36.8) | 34 (29.6) | 0.22 |
| SOFA score without respiratory item | 5.0 [3.0, 8.0] | 5.00 [3.0, 8.0] | 5.00 [3.0, 7.0] | 6.0 [4.0, 8.0] | 0.12 |
| Use of vasopressor | 39 (28.3) | 108 (34.4) | 37 (27.2) | 41 (35.7) | 0.58 |
| PaO_2_/FiO_2_ ratio , mmHg | 161.4 [101.2, 211.7] | 161.4 [119.5, 240.9] | 161.4 [116.6, 216.0] | 162.5 [113.2, 252.0] | 0.43 |
| Oxygen flow, l/min | 6.0 [3.0, 10.0] | 5.00 [3.0, 9.0] | 6.00 [3.0, 12.0] | 5.00 [2.0, 10.0] | 0.96 |
| Glasgow coma score | 15.0 [15.0, 15.0] | 15.0 [15.0, 15.0] | 15.0 [14.0, 15.0] | 15.0 [13.0, 15.0] | 0.03 |
| Respiratory rate>30/min | 84 (60.9) | 190 (60.5)) | 88 (64.7) | 55 (47.8) | 0.14 |
| Number of quadrants on chest X-ray | 2.0 [1.0, 4.0] | 2.0 [1.0, 4.0] | 2.0 [1.0, 3.0] | 2.0 [1.0, 4.0] | 0.50 |
| **Outcome** |  |  |  |  |  |
| ICU mortality | 39 (28.3) | 94 (29.9) | 44 (32.4) | 51 (44.3) | 0.006 |
| Hospital mortality | 53 (38.4) | 131 (41.7) | 66 (48.5) | 59 (51.3) | 0.01 |

Values are given in N (%) or median [IQR]. Definition of abbreviations: IQR = Interquartile Range; ICU = Intensive Care Unit; SOFA = Sequential Organ Failure Assessment

**Table S-4.**  Center characteristics by quartiles of ICU adjusted rates of invasive mechanical ventilation-TRIAL-OH cohort (n=17)

|  | | **Quartile 1**  **(Lowest)** | **Quartile 2** | **Quartile 3** | **Quartile 4 (Highest)** | **p-value** |
| --- | --- | --- | --- | --- | --- | --- |
| **Intubation rate, %** | | 44 | 55 | 60 | 72 | <0.001 |
| **No. of beds (ICU)**  **median [IQR]** | | 40.0 [18.0, 44.0] | 18.0 [14.0, 21.2] | 29.50 [22.0, 46.2] | 17.0[16.0, 23.7] | 0.57 |
| **No. of beds (hospital)** | 1099.0[1060.0, 1551.0] | | 465.5 [270.7, 793.7] | 1324.5 [1112.5, 1586.7] | 805.5 [658.2, 975.0] | 0.81 |
| **Hematological ward in hospital** | | 5 (100.0) | 4 (100.0) | 4 (100.0) | 3 (75.0) | 0.16 |
| **No. of ID patients/ years** | | 70.0 [69.0, 71.0] | 97.5 [37.7, 164.7] | 80.0 [61.0, 95.0] | 43.0 [30.7, 52.5] | 0.28 |
| **No. of patients/ years,** | | 872.0 [800.0, 950.0] | 507.0 [342.5, 698.0] | 876.0 [619.0, 1220.0] | 561.0 [507.5, 621.7] | 0.32 |
| **No. of IMV patients/ years** | | 506.0 [336.0, 576.0] | 225.0[143.2, 352.2] | 375.0 [372.5, 412.5] | 407.0 [386.0, 421.5] | 0.87 |
| **University hospital, n (%)** | | 4 ( 80.0) | 2 ( 50.0) | 4 (100.0) | 3 (75.0) | 0.75 |
| **Annual IMV rate** | | 58.0 [42.0, 58.2] | 42.2 [36.9, 54.3] | 57.5 [49.2, 64.3] | 71.2 [66.8, 71.4] | 0.08 |

Values are given in N (%) or median [IQR]. Definition of abbreviations: IQR = Interquartile Range; ICU = Intensive Care Unit; SOFA = Sequential Organ Failure Assessment; ID= immunocompromised patients; IMV= invasive mechanical ventilation

**Table S-5**. Patient characteristics by quartiles of ICU adjusted rates of invasive mechanical ventilation-HIGH-trial (n=776)

|  | **Quartile 1**  **(Lowest)** | **Quartile 2** | **Quartile 3** | **Quartile 4 (Highest)** | **p-value** |
| --- | --- | --- | --- | --- | --- |
| **Intubation rate, %** | 28 | 35 | 47 | 53 | <0.001 |
| **Characteristics of the patients** |  |  |  |  |  |
| Age, median [IQR], year | 64.0 [56.0, 71.0] | 65.0 [57.2, 73.0] | 62.0 [53.0, 69.0] | 65.0 [58.0, 71.0] | 0.73 |
| Male sex, n (%) | 70 (30.7) | 34 (32.1) | 90 (36.7) | 65 (33.0) | 0 .40 |
| Charlson score | 5.0 [4.0, 7.5] | 5.0 [4.0, 6.0] | 4.00 [3.0, 6.0] | 5.0 [4.0, 8.0] | 0.21 |
| Respiratory comorbidity | 76 (33.3) | 27 (25.5) | 82 (33.5) | 57 (28.9) | 0.27 |
| Hematologic malignancy | 85 (37.3) | 47 (44.3) | 132 (53.9) | 84 (42.6) | 0.07 |
| Allogeneic stem cell transplantation | 15 ( 6.6) | 6 ( 5.7) | 28 (11.4) | 12 ( 6.1) | 0.8 |
| Poor performance status (>2) | 98 (43.0) | 45 (42.5) | 73 (29.8) | 66 (33.5) | 0.01 |
| Time from respiratory symptoms to ICU admission, day | 1.0 [0.0, 3.0] | 0.0 [0.0, 2.0] | 0.0 [0.0, 1.0] | 0.0 [0.0, 1.0] | <0.001 |
| **Clinical parameters at baseline** |  |  |  |  |  |
| Neutropenia | 26 (11.4) | 20 (18.9) | 50 (20.4) | 40 (20.3) | 0.01 |
| SOFA score without respiratory item | 3.00 [3.0, 4.0] | 3.50 [3.0, 4.0] | 3.00 [3.0, 4.0] | 3.00 [3.0, 4.0] | 0.77 |
| Use of vasopressor | 37 (16.2) | 18 (17.0) | 61 (24.9) | 37 (18.8) | 0.14 |
| PaO_2_/FiO_2_ ratio , mmHg | 161.4 [101.2, 211.7] | 161.4 [119.5, 240.9] | 161.4 [116.6, 216.0] | 162.5 [113.2, 252.0] | 0.43 |
| Oxygen flow, l/min | 10.0 [6.0, 15.0] | 12.0 [6.75, 15.0] | 12.0 [6.0, 15.0] | 10.0 [6.0, 15.0] | 0.45 |
| Glasgow coma score | 15.0 [15.0, 15.0] | 15.00 [15.0, 15.0] | 15.00 [15.0, 15.0] | 15.0 [15.0, 15.0] | 0.63 |
| Respiratory rate>30/min | 142 (62.3) | 61 (57.5) | 141 (57.6) | 120 (60.9) | 0.57 |
| Biliterate infiltrates on chest X-ray | 119 (52.2) | 59 (55.7) | 172 (70.2) | 124 (62.9) | 0.26 |
| **Outcome** |  |  |  |  |  |
| ICU mortality | 66 (28.9) | 31 (29.2) | 69 (28.2) | 79 (40.1) | 0.05 |
| Hospital mortality | 94 (41.2) | 36 (34.0) | 96 (39.2) | 96 (48.7) | 0.34 |

Values are given in N (%) or median [IQR]. Definition of abbreviations: IQR = Interquartile Range; ICU = Intensive Care Unit; SOFA = Sequential Organ Failure Assessment

**Table S-6**. Center characteristics by quartiles of ICU adjusted rates of invasive mechanical ventilation-HIGH Study (n=31)

|  | **Quartile 1**  **(Lowest)** | **Quartile 2** | **Quartile 3** | **Quartile 4 (Highest)** | **p-value** |
| --- | --- | --- | --- | --- | --- |
| **Intubation rate, %** | 28 | 35 | 47 | 53 |  |
| **No. of beds (ICU)**  **median [IQR]** | 19.0 [16.5,21.5 ] | 28.0 [18,32 ] | 20.5 [16.0,25.0 ] | 24.0 [10.5, 30.0] | 0.84 |
| **No. of beds (hospital)** | 1200.0 [600.0, 1200.0] | 1078.5 [993.5, 1185.5] | 850.0 [687.5, 1125.0] | 875.0 [796.6, 1049.3] | 0.54 |
| **Hematological ward in hospital** | 7 (87.5) | 4 (50.0) | 5 (71.4) | 6 (75.0) |  |
| **No. of ID patients/ years** | 200.0 [75.0, 250.0] | 67.5 [33.6, 172.5] | 95.0 [57.5, 177.5] | 100.0 [62.5, 139.8] | 0.62 |
| **No. of patients/ years,** | 1100.0 [1000.0, 1500.0] | 1100.0 [700.0, 1300.0] | 650.0 [540.0, 887.5] | 911.0 [488.0, 1137.5] | 0.28 |
| **No. of MV patients/ years** | 300.0 [30.00, 675.0] | 475.0 [337.5, 607.5] | 380.0 [370.0, 500.0] | 472.5 [243.6, 682.5] | 0.39 |
| **University hospital** | 7 (87.5) | 5 (62.5) | 6 (85.7) | 6 (75.0) | 0.82 |
| **Annual IMV rate** | 32.7 [30.0, 45.0] | 53.6 [49.2, 57.9] | 60.0 [50.0, 71.1] | 56.3 [48.2, 59.2] | 0.01 |

Values are given in N (%) or median [IQR]. Definition of abbreviations: IQR = Interquartile Range; ICU = Intensive Care Unit; SOFA = Sequential Organ Failure Assessment; ID= immunocompromised patients; IMV= invasive mechanical ventilation

**Figure S-3.** Ranking of ICUs by center effect on the mean intubation risk with and without adjustment on annual invasive mechanical ventilation rate and time from respiratory symptoms to ICU admission


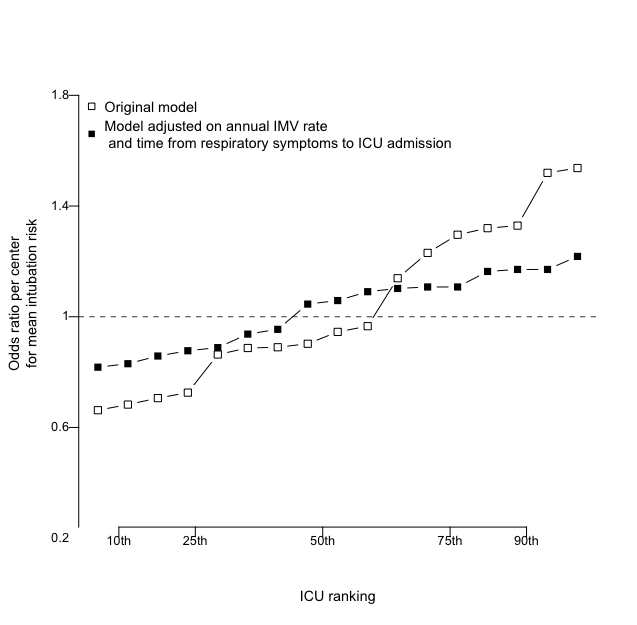

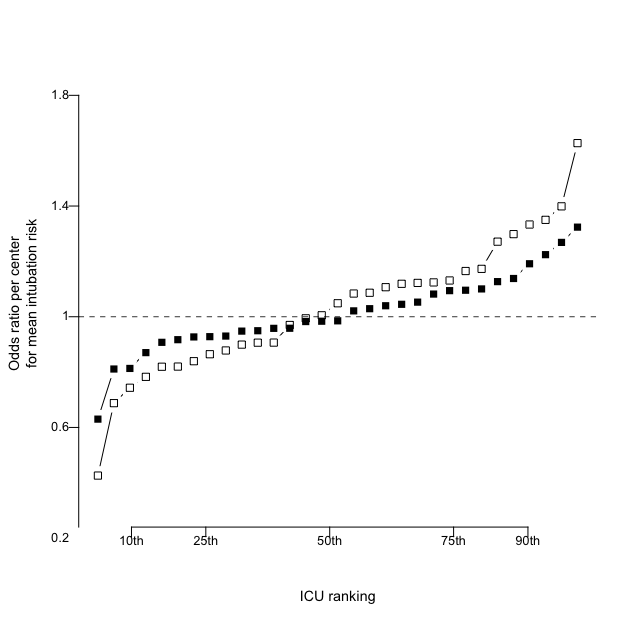


**HIGH trial**

**TRIAL-OH Cohort**

Squares represent center effects on the mean intubation risk as odds ratio (OR) (comparison of each center to a theoretical average reference center with OR = 1). x-axis gives the percentiles of the distribution of center effect on mean intubation risk.

The white squares are effects in the multivariate model adjusted on individual prognostic factors alone and black ones represent center effects when the model is further adjusted on the IMV rate and time from respiratory symptoms onset to ICU admission (p-value for center effect, Trial-OH cohort: 0.5, High trial: 0.1).

1. **Sensitivity analyses**

**Figure S-4.** Center effect on the effect of oxygenation strategy (High Flow Nasal cannula) on Intubation risk in the High Trial


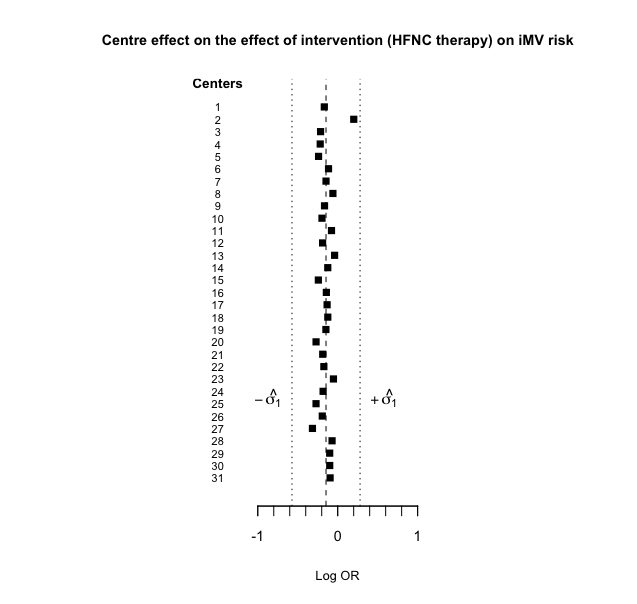


Centers are sorted by size. Black squares represent the adjusted estimated of each center. Abbreviations : OR : Odds ratio

**Results:**

As shown, there was little variation around the mean effect.

Permutation test for center effect on the mean intubation rate: p-value= 0.037

Permutation test for between-center heterogeneity of the effect of HFNC use on subsequent intubation risk: p-value= 0.188

1. **References**

1. Merlo J, Chaix B, Ohlsson H, et al (2006) A brief conceptual tutorial of multilevel analysis in social epidemiology: using measures of clustering in multilevel logistic regression to investigate contextual phenomena. J Epidemiol Community Health 60:290–297. https://doi.org/10.1136/jech.2004.029454

2. Austin PC, Merlo J (2017) Intermediate and advanced topics in multilevel logistic regression analysis. Stat Med 36:3257–3277. https://doi.org/10.1002/sim.7336

3. Glidden DV, Vittinghoff E (2004) Modelling clustered survival data from multicentre clinical trials. Stat Med 23:369–388. https://doi.org/10.1002/sim.1599

4. Kahan BC (2014) Accounting for centre-effects in multicentre trials with a binary outcome - when, why, and how? BMC Med Res Methodol 14:20. https://doi.org/10.1186/1471-2288-14-20

5. Drikvandi R, Verbeke G, Khodadadi A, Partovi Nia V (2013) Testing multiple variance components in linear mixed-effects models. Biostat Oxf Engl 14:144–159. https://doi.org/10.1093/biostatistics/kxs028

6. Biard L, Darmon M, Lemiale V, et al (2019) Center Effects in Hospital Mortality of Critically Ill Patients With Hematologic Malignancies. Crit Care Med Online First: https://doi.org/10.1097/CCM.0000000000003717

7. Fitzmaurice GM, Lipsitz SR, Ibrahim JG (2007) A note on permutation tests for variance components in multilevel generalized linear mixed models. Biometrics 63:942–946. https://doi.org/10.1111/j.1541-0420.2007.00775.x

8. Biard L, Porcher R, Resche-Rigon M (2014) Permutation tests for centre effect on survival endpoints with application in an acute myeloid leukaemia multicentre study. Stat Med 33:3047–3057. https://doi.org/10.1002/sim.6153
